# Addressing the translation gap in SCT donor selection

**DOI:** 10.3389/fimmu.2026.1861400

**Published:** 2026-06-24

**Authors:** David Higgins, Madlen Reschke, Jonas Seiler, Jonathan P. Gross, Lena Oevermann

**Affiliations:** 1 Berlin Institute of Health at Charité - Universitätsmedizin Berlin, Berlin, Germany; 2 Department of Pediatric Oncology and Hematology, Charité – Universitätsmedizin Berlin, corporate member of Freie Universität Berlin, Humboldt – Universität zu Berlin, and Berlin Institute of Health, Berlin, Germany; 3 German Center for Child and Adolescent Health (DZKJ) - Berlin Site, Berlin, Germany

**Keywords:** adult SCT, aGVHD, donor selection, GRFS, human stem cell transplantation (HSCT), machine learning, pediatric SCT, relapse

## Abstract

Machine learning (ML) offers genuine promise for improving donor selection in hematopoietic stem cell transplantation (SCT), yet the gap between model development and clinical adoption remains wide. Drawing on experience from a German federally-funded translational incubation project at the Berlin Institute of Health and Charité Hospital (2021-2023), we offer a practitioner’s perspective on what it actually takes to move ML-driven SCT tools from research into clinical practice. We make two distinct contributions. First, we offer concrete prescriptions on data strategy and translational methodology: registry data was not designed for ML and must be rethought from the ground up; multi-center data infrastructure is a prerequisite, not an aspiration; approaches which increase the breadth of clinical parameters are theoretically sound and should be embraced; and the field must move beyond retrospective validation toward prospective studies designed around standard-of-care endpoints. Second, we provide deliberate sensitization to three forces that will surprise clinically-focused teams: the product and regulatory burden of sustaining a clinical tool; the true resource requirements for the full translational path; and the market dynamics and misaligned institutional incentives that impede adoption. Despite these barriers, the technical feasibility of ML-supported SCT donor selection is real, and the community’s demonstrated willingness to share data provides the foundation on which to build. What is needed now is not more retrospective modelling, but projects that enter the space with a clear-eyed understanding of the full landscape.

## 1 Introduction

Despite a growing body of machine learning (ML) research in hematopoietic stem cell transplantation (SCT), ML-supported donor selection has yet to make a meaningful impact on clinical practice (1). The science is advancing; the translation is not.

Examples of scientific success include predicting post transplant adverse outcomes using donor registry data (2–6). However, performance tends to plateau at clinically unacceptable levels, and pleas are regularly made for recording of additional input parameters (7). Avoiding the issue of difficult to curate, broadly defined clinical input parameters, specialist models focus on core aspects of the matching process such as Predicted Indirectly ReCognizable HLA Epitopes (PIRCHE) (8–10), Killer Cell Immunoglobulin-like Receptor typing (KIR) (11, 12) and the HLA-DPB1 Algorithm (13). Meanwhile, the Endothelial Activation and Stress Index (EASIX) continues to show value as a stratification tool with potential for predicting certain post-transplant outcomes (14), even when evaluated post-transplantation (15). Finally, focused work on pre-transplantation donor cell mobilization (16–18) and post-transplantation biomarkers (19), show potential for allowing clinicians to make early protocol-change decisions, with the goal of influencing clinical outcomes.

From the end of 2021 until the end of 2023 we pursued a translational incubation project funded by the German federal government (BMBF) via the Berlin Institute of Health and Charité Berlin, with the purpose of developing an ML-driven tool to better predict clinical outcomes - initially acute Graft-vs-Host-Disease (aGvHD) - at the time of donor selection. The scientific outcomes of that project, covering both pediatric and adult SCT recipients across a massively multi-centric international cohort, are reported in a companion research manuscript. Here we report something different: what we learned about the gap between developing such a tool and actually delivering it into clinical practice.

This perspective is addressed to researchers and clinicians who are considering, or already pursuing, data-driven approaches to SCT. We offer both concrete prescriptions - on data strategy, data infrastructure, and translational methodology - and deliberate sensitization to the product, resource, and market realities that derail technically sound projects. Our central argument is simple: translation is not an extension of research. It is a distinct set of activities, and it must be planned and resourced as such from the outset.

## 2 Data

Data is gathered for a purpose. Frequently uttered as a performative maxim, rather than systematically implemented, this remains a principle of sound data-driven science. Our advice reads, “If you really want the data to be *effective* for a given purpose, it is usually better to redesign the data gathering processes with a clear objective.

With this in mind, registry data has the purpose of supporting existing, and historic, donor matching processes. It is the best system we have, for a process which was designed when large-scale data bases had 1000s of entries - not the millions of entries which any modern laptop can handle with ease. The actual donor selection is largely a manual, clinician-scientist driven process, missing out on the opportunities for large-scale automation provided by modern computing.

Adding rows of data, i.e. more patients, is the best known practice for improving the quality of prediction models (20). Sometimes called ‘tall data’ this is the basis for the original big data revolution (c. 2006). More data drives better model performance up until the limits of the signal present in the data (21). But remember, SCT is not a widely performed technique (22, 23), and the standard of care has a tendency to change more quickly than sufficient data can be gathered in this field. The issue here is that, any ML model trained on a previous standard-of-care will not work under new clinical practices.

Traditional statistical training focuses on low-dimensional input analyses, and warns against the risks associated with the addition of extra clinical parameters to an analysis (e.g. spurious correlations). Less widely known is the degree to which ML has long moved beyond a low-dimensional analytic world, and there are strong theoretical foundations to this move (24–26). Handled correctly, ML thrives in a high input-dimension regime, accurately identifying complex input-output relationships. However, the correct handling here imposes strong methodological constraints, which in turn impact deeply on both the data gathering and model validation processes (27).

The risks posed by developing a ML solution naively are well understood in the literature (28, 29). There is no turnkey method which will safely model clinical data accurately. But methods such as rigorous validation, especially out-of-distribution testing in multicenter settings followed by systematic investigation of the underlying distributional shifts, if required, are a must. Clinical reasoning to constrain the feature set *a priori*, rather than letting the model fish freely across all available variables, and harmonized data collection processes across sites where feasible, are easily understandable and plannable by an expert operator. Here we need to adapt our team skills to include considerably more expertise in ML and ML validation.

In contrast to expanding the input parameter dimensions to incorporate multiple types of clinical variable, specific matching tools typically require narrowly defined input parameters. Such tools will always outperform generic tools - on the defined specific task. Good examples here include PIRCHE (8–10) and KIR (11, 12, 30) matching. It is technically challenging to build a generic matching tool which can perform as well, on these narrow tasks, as the specialist tools do. Conversely, it is relatively easy for a general matching tool to incorporate PIRCHE and KIR match scores as new and/or optional inputs in solving a broader matching problem.

Specific matching tools are good for their specific issue, but there are a lot of reasons for a (SCT) graft not to work (31). This means, to improve SCT outcomes one would need many specific models. The history of AI is littered with dead specific-model projects which were superseded by much simpler approaches which had access to the right data (32, 33).

Our project was partially impacted by the change in standard of care, to incorporate PT-Cy/Abatacept conditioning regimen. Looking at the history of SCT these revolutionary changes to standard of care emerge with satisfying regularity.

To name just three currently emerging technologies: The 2025 annual meeting of the American Society of Hematology (ASH) demonstrated considerable progress in using monoclonal antibody therapies for many of the underlying conditions currently treated by SCT (34–36); In our clinical practice, we observe the rise of haplo-matched SCT, including ex-vivo T-cell depletion, and new prophylaxis such as Roxolitinib (37); Immune cell repertoire screening technologies are continuing to advance, giving access to emerging - potentially paradigm changing (38, 39) - methods of matching donors to recipients. But, realistically, SCT will not go away any time soon. So better donor selection will remain a clinical challenge for many years to come. In which case, designing a system which can cope with these changes to clinical care is vital.

**Figure 1:**
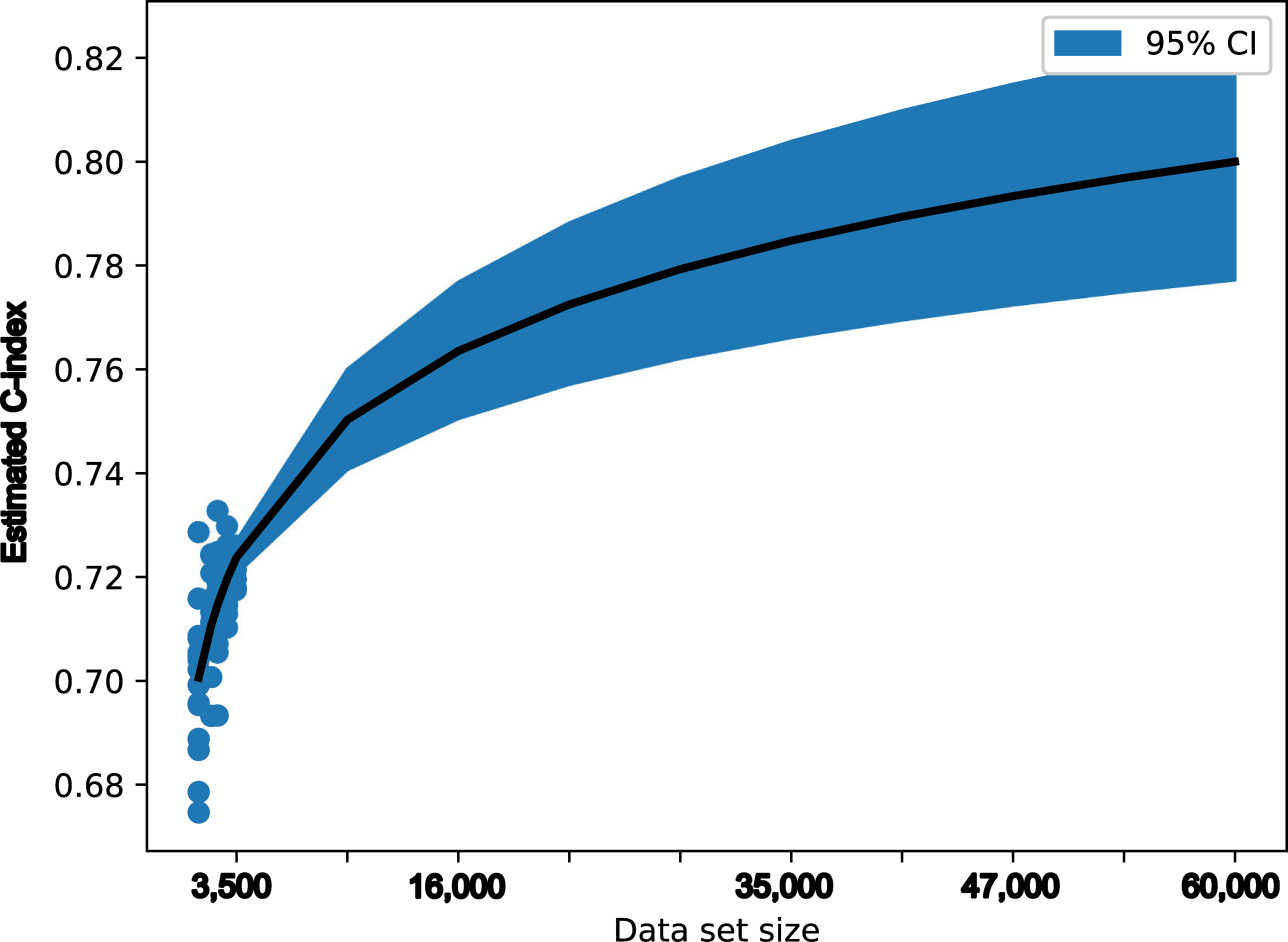
A log-fit (r = 0.68) extrapolation from n = 3,534 points shows we would need 59,569 (28,310 - 206,068; 95% confidence) data points to achieve a c-index = 0.8.

Each of the above points impose massive structural constraints on both the data gathering and model performance validation activities of the project. Happily, there are sufficient SCTs taking place across the community that a multi-center collaborative approach remains technically feasible. **Figure 1** shows the estimated number of training data points required to attain receiver operating characteristic (ROC) performance of 0.8 on aGvHD prediction, using the method of Rosenfeld (40), is approximately 60,000 transplants. Taking this into account, we believe that it is possible to design a data gathering process from scratch with the goal of supporting SCT donor selection on an ongoing basis.

Our advice is to redesign data acquisition with the end purpose in mind. The process needs to be multi-center, not just during model building and validation but throughout the operational lifetime of the model in order to keep up with changes in care. Remember changes in care typically happen on a center-by-center and clinician-by-clinician basis, not all-at-once or incrementally. Capturing additional clinical parameters is likely to pay off over time, as long as they are handled, not naively, but following ML best-practices (27). Focusing on a narrow single-cause of graft rejection is moving the problem from one of data acquisition to one of multiple model creation; this is not a solution. Here we lean firmly in the direction of developing a comprehensive solution, and modeling the interplay of complex and competing outcomes. Finally, design the prediction tool in a manner which incentivizes users to also input, and share, outcomes data; without this, again, the system will fail to stay up to date.

## 3 Translation as a set of activities

Research areas, which show success in translation (41–44), tend to be fields such as genomics (45, 46), where the output of scientific discovery (e.g. a gene-disease association) is seen as a direct input for large-scale industrialization processes, such as pharma R&D (47). In these fields, the path from discovery to clinical impact is well-trodden, heavily resourced, and structurally incentivized. In our field, in contrast, there is a behavioral gap between the output of the research results and adoption in the clinic (48).

The evidence-based medicine movement has a strong point in their complaints that too many modern clinical studies are focused on (re-)evaluating existing standards of care, and do not focus sufficiently on clinical endpoints with a clear potential to change standards of care (49, 50). Data and ML projects, necessitating heavily data acquisition focused initial-stages seem to be particularly backward-facing in their approaches to validation. They rarely seem to proceed to the endpoints required to validate potential changes to standards of care.

While it will always be necessary to use retrospective data, in order to build confidence for a more expensive prospective study, the introduction of Data and AI/ML to clinical care entails changes in practices and cannot be exclusively evaluated retrospectively. Clinicians and researchers must engage in more prospective studies of AI/ML driven innovation, and pay careful attention that the endpoints support changes in clinical care rather than focusing on so-called ‘safe’ outcomes.

If we want to see our work appearing in clinical practice, we must also engage in deliberate Acts of Translation, such as our own incubation project. The most interesting work being published is always going to be Research work - but, direct research outputs are difficult to synthesize into coherent outcomes-driven changes to clinical practices. Translation is not a downstream consequence of good research. It must be planned, resourced, and pursued as a distinct activity in its own right.

## 4 The product mindset

A number of successful clinical innovation programs have been developed globally (51–53). The repeated experience of both Clinicians and Researchers participating in these structured programs is an overwhelming shock at the number of steps and behind the scenes processes necessary to maintain even the simplest of clinical products (54). Understanding this issue is the first step in being able to plan for and deal with it.

To be useful in clinical practice a tool must be Safe, Effective, and Available. In practice, this means that a clinical tool is and will always be a product. A product is not a one-time solution. Nor can it depend upon the extraordinary contributions of a uniquely skilled expert researcher. While expertise is a prerequisite for anything in clinical practice, uniqueness is a barrier to scaling to other patients in a reliable and reproducible manner.

## 5 Resources: team, time and money

Specialist skill sets are required to work in both computational immunology and SCT. The standard of care is regularly changing, and appears to be changing again right now (see our comments in the section on Data).

The breadth and depth of skills required to bring a medical AI product - such as a SCT donor selection tool - to the clinic is particularly unusual. A product is not research, and research scientists in particular tend to struggle to make the jump to clinical product development. On the technical side, patient safety is the driving factor. This is evaluated via the regulatory compliance process, and is best implemented by a team which contains at least one technical professional with strong software as a medical device (SaMD) experience. Regulatory expertise itself can largely be bought-in on a part-time basis. However, teams need to understand that regulatory serves primarily as a filter, preventing unsafe products reaching the market, rather than as a clear roadmap leading towards success. Clinician inputs are vital for the earliest stages of product development, and a background level of clinician oversight remains important throughout the product lifecycle. For SCT donor selection, in particular, it is important to buy clinicians out of their clinical time, while enabling them to continue to work in elite transplantation units. Balancing clinician availability is both necessary and extremely complicated.

Ultimately, for a clinical AI tool to make it from research all the way to clinical practice requires multiple clinical studies and a detailed regulatory strategy. Despite working in a relatively low-cost setting (Europe) vis-a-vis our initial target market (USA), we estimated a budget of €12-15m would be required to get our tool all the way through regulatory and into the market. This budget is dominated by the long time periods involved, in which a product must first be prepared to a level of performance and safety required, prior to evaluating it in a clinical study; and the study period itself which, of necessity, may take 3+ years to recruit sufficient numbers of patients and allow for appropriate follow-up periods. These budget numbers may be deceptive given what we read about specific programs and therapies in cancer care. From the perspective of transforming SCT they are a heavy lift, and would only be possible by leveraging personal networks rather than investor greed.

## 6 Market: access, vested interests, mis-aligned interests

The frequency distribution of stem cell transplants follows a skewed distribution. According to US Health Resources & Services Administration (HRSA) data, a handful of centers carry out the majority of transplants (“Transplant Activity Report | Blood Stem Cell,” 2019). However, no single center carries out sufficient number of transplants necessary to safely build and validate a donor selection tool [c.f. **Figure 1]**.

The presence of pharma money in oncology posed both an opportunity and a hurdle throughout our project. On the one hand, it is a clear enabler - investigator initiated studies (IIS) are widely supported and can be well funded. On the other hand, a minority of researchers and clinicians directly expressed skepticism about our motivations and were of the opinion that we would steal patient data and make ourselves rich in the process. While we agree that a degree of such skepticism is healthy, the black-and-white nature of it makes progress impossible. Overall our advice is to be aware that the presence of pharma research money is now deeply ingrained in clinical operations and validation studies must be planned in a manner which does not threaten the business model of the clinics.

During the lifetime of our project, most major US healthcare providers announced funding on the order of $50m per institution to develop internal AI offerings across the entirety of their healthcare programs. This acted as an additional barrier to rolling out collaborations. Each center was individually incentivized to first try building their own solutions before, we expect, coming a few years later to the realization that only a community pooling of data can work in the case of SCT donor selection.

These issues add up, through what business-side people call variously Access Issues, Mis-aligned Interests and Vested Interests, to create serious impediments to delivery of clinically impactful innovative data projects. Similarly to the issues of Team, Time and Resources, a clinically focused team is likely to face many unwelcome surprises on this front. The only way to overcome them is to identify these issues early and to systematically mitigate each of them.

## 7 Conclusion

This perspective has two distinct purposes, and it is worth being explicit about both.

On the question of data, we have offered concrete prescriptions: redesign data acquisition with the end purpose in mind; build multi-center infrastructure from the outset; embrace broader parameter sets while following ML best-practices; and design the prediction tool itself to incentivize ongoing outcomes sharing. These are not obvious choices for a community trained in classical statistical approaches to clinical data, and getting them wrong early forecloses options later. The same applies to our argument on Translation - the field must move beyond retrospective validation and commit to prospective studies designed around standard-of-care endpoints. This is where the scientific contribution lies, and where we believe the community can act.

On the questions of Product, Resources, and Market, our intent is deliberately different. These are not areas where clinicians and researchers need a technical manual - the relevant literature exists and is cited here. What they need is early warning. The repeated experience of structured clinical innovation programs is that technically excellent teams are blindsided by the complexity of building and sustaining a clinical product, by the true cost of the regulatory path, and by the misaligned incentives that make clinical deployment harder than the science. Forewarned is not the same as forearmed, but it is a necessary precondition.

Taken together, we believe a coherent roadmap for computationally-supported SCT donor selection is achievable. The community’s generosity in sharing data - which made our own project possible - suggests the will is there. What remains is to ensure that the next generation of projects enters this space with a clearer understanding of the full landscape, not just the science.

## Data Availability

This article is a Perspective, the major aspects of which are not dependent on Data. **Figure 1** was prepared, for the convenience of the audience, from original data. This data is original patient data from approximately 6,000 patients at 8 institutions worldwide. A detailed report into this data, including presentation of predictive models based upon this data will be presented in a separate Research manuscript. Access to the original data is subject to individual permissions from the originating institutions, and as original patient data such permission is difficult to obtain and subject to many institution specific permissions. As senior author on both this manuscript and on the upcoming related Research manuscript Dr. Lena Oevermann is the initial point of contact for coordinated access requests to the underlying data (Email: lena.oevermann@charite.de).
